# Comprehensive global review and methodological framework for developing food atlases

**DOI:** 10.3389/fnut.2024.1505606

**Published:** 2024-11-22

**Authors:** Ghadir Fallata, Rasil Alhadi, Luluh Alnashmi, Shahad Aljohani, Fatimah Alsaeed

**Affiliations:** ^1^Saudi Food and Drug Authority, Riyadh, Saudi Arabia; ^2^Department of Community Health Sciences, College of Applied Medical Sciences, King Saud University, Riyadh, Saudi Arabia; ^3^Department of Health Sciences, College of Health and Rehabilitation Sciences, Princess Nourah Bint Abdul Rahman University, Riyadh, Saudi Arabia; ^4^Department of Applied Medical Sciences, Imam Abdulrahman bin Faisal University, Dammam, Saudi Arabia

**Keywords:** food atlas, atlas, global food, review, food consumption

## Abstract

**Introduction:**

An atlas is a trustworthy resource created from precise data collection that serves as a guide for communities. A food atlas is a useful tool for analyzing dietary data. There is a growing need for a food atlas that is part of the nation’s strategy to help the health sector with specific nutritional or dietary assessments of individual consumption and overall wellbeing. Although researchers previously attempted to create a food atlas on a national level, the process of data collection was not well defined.

**Methods:**

This study provides an overview of global food atlases that can be used to develop a procedure manual to guide experts in creating a dependable food atlas.

**Results:**

To date, 27 countries have developed food atlases for various reasons. After examining these countries’ experiences, six important steps in the procedure manual that should be considered when developing a food atlas were identified: choosing the most consumed food, using traditional cooking utensils, determining portion sizes, capturing photographs of the food, validating the food atlas, and publishing the food atlas.

**Discussion:**

This procedure manual can be used as a guide until a validation study is conducted.

## 1 Introduction

A food atlas is a visual guide that illustrates common foods or dishes from a population’s national or regional diet, along with typical serving sizes (1). The purpose of a food atlas is to accurately determine portion sizes, which are crucial for understanding the nutritional value of food. Several food atlases have been developed, each with a specific focus. For instance, Greece created its first food atlas in 1992, featuring 170 different foods (2). The Northern Italy atlas, developed in 2005, includes the highest number of foods, totaling 434 items (3). Conversely, some countries may not have a food atlas owing to several reasons. One possible reason is limited financial and technical resources. Additionally, in some developing countries, the absence of infrastructure and trained personnel capable of collecting, analyzing, and maintaining data on food consumption patterns may pose challenges. Cultural and regional diversity in dietary practices further complicates efforts to standardize portion sizes and food descriptions. Moreover, the lack of interdisciplinary collaboration among nutritionists, public health experts, and other professionals hinders the development of accurate and culturally specific food atlases (4).

Various types of atlases exist, each with its own objective. Atlases may focus on nutrition, agriculture, specific diseases, age groups, and public health policies or nutrition interventions (5, 6). The most well-known type of atlas is the food atlas, which features various photographs of dishes. For example, the United Kingdom (UK) food atlas was created to assess children’s nutritional consumption (6), whereas Ecuador’s food atlas was designed for adults (7). Additionally, atlases created for specific diseases, such as dysphagia, have been used as valuable tools by healthcare professionals to educate patients and caregivers about the required texture-modified food and thickened fluid (5). Moreover, several existing food atlases have profoundly impacted public health policies and nutrition interventions by supplying critical data on food availability, consumption trends, and dietary diversity, which facilitate evidence-based policymaking. For example, the Food Atlas for the USA has been instrumental in identifying food deserts and devising targeted strategies to enhance access to nutritious foods, thereby influencing local and national policies aimed at mitigating diet-related health issues (4). Likewise, food atlases in developing countries have uncovered nutritional deficiencies and directed efforts to improve food security through agricultural and health-related programs (4).

In food atlases, data are typically collected from sources such as Food Frequency Questionnaires (FFQs), surveys, 24-h recalls, restaurant menus, household surveys or home visits, recipe books, and other sources (8–11). In most countries, 24-h Dietary Recall and FFQs are used to estimate food intake; however, these methods are often inaccurate because they rely on the respondents’ short-term memories and the interviewer’s prior experience. To determine the portion size of a dish, weighing the food before and after consumption is considered the most accurate measurement method. However, this approach is not suitable for dietary surveys involving many people because it is time-consuming and has a significant respondent load. Visual aids, such as plastic food replicas (food models) and photographs, may help to reduce errors made while estimating food portions during dietary recall. The accuracy of food-portion measurements in dietary recalls can be improved by using pictures.

Furthermore, a previous study found that two-dimensional images could accurately describe food portions in a manner comparable to three-dimensional food models. Many people and nations have investigated the reliability of using food images to estimate portion sizes. Their findings confirm the value of using food images to estimate serving size. Adopting precise food portion sizes suited to the local context is crucial for nutritional assessments because eating patterns differ between nations (12). Additionally, food is grouped into different categories in the atlas; for example, the food group is based on the most consumed food by society (e.g., how food is collected in Lebanon) and shows varied portion sizes to help estimate consumption, as well as inform consumers about the nutritional value and portion sizes (10).

At the national level, the Saudi food atlas was established in 2018 by the University Center at the College of Medicine at King Saud University owing to the need to consider one of the most critical health problems both globally and in the Kingdom of Saudi Arabia (SA): obesity (13). The General Authority for Statistics announced a bulletin of indicators of health determinants in SA for the year 2023 on its official website; the results of the bulletin stated that the prevalence of obesity among the adult population of SA reached 23.7%. This percentage was similar between males and females, whereas the percentage of those with ideal weight was significantly higher among females (39.6%) than males (29.5%). Among children aged <15 years, the prevalences of obesity and underweight were 7.3 and 41%, respectively. SA exhibits one of the highest prevalence rates of overweight and obesity among all age groups and children; thus, considering that the population is at significant risk for increased rates of noncommunicable disease mortality, these prevalences have also increased rapidly over the past few decades (14).

Food atlases serve various purposes, including health and nutrition; using effective tools, food atlases serve as a valuable resource for researchers conducting surveys. They also provide accurate information for implementing nutritional interventions. Although some researchers attempted to develop a food atlas at the national level, the methods used to collect information were unclear, and it is considered necessary to obtain a comprehensive understanding of the global food atlas and replicate the general steps taken to develop a national food atlas that reflects individual consumption patterns. This global assessment compiles general food atlases created for healthy adults worldwide to assist future researchers, sectors, and organizations in updating or developing food atlases.

### 1.1 Objective and scope

The objective of this study was to provide a descriptive global review of food atlases (the main reasons for creating and using food atlases, photography, portion size, number of food items, and how consumption information was collected for all food atlases around the world) that covers all adult food atlases published between 1992 and 2020. Additionally, this study aimed at establishing a procedural manual or criteria to support the development of national food atlases.

## 2 Materials and methods

This narrative review was conducted by collecting published scientific literature on food atlases from PubMed and Google databases in August 2021. The PubMed database was used to extract studies using keywords related to food and world atlases. A total of 682 studies were extracted from PubMed, 15 of which were included in the review after the data were screened according to predefined inclusion and exclusion criteria. The inclusion criteria for the review were studies related to food atlases (including topics such as the most famous food, photos, information about multiple portion sizes, and use of appropriate cutlery), and those including healthy adults (aged ≥18 years, both sexes). Moreover, to incorporate a food atlas from each country, the “Google” search engine was used to ensure that all countries were included. Generally, both methods were used to search for the food atlases in countries worldwide.

**Figure 1:**
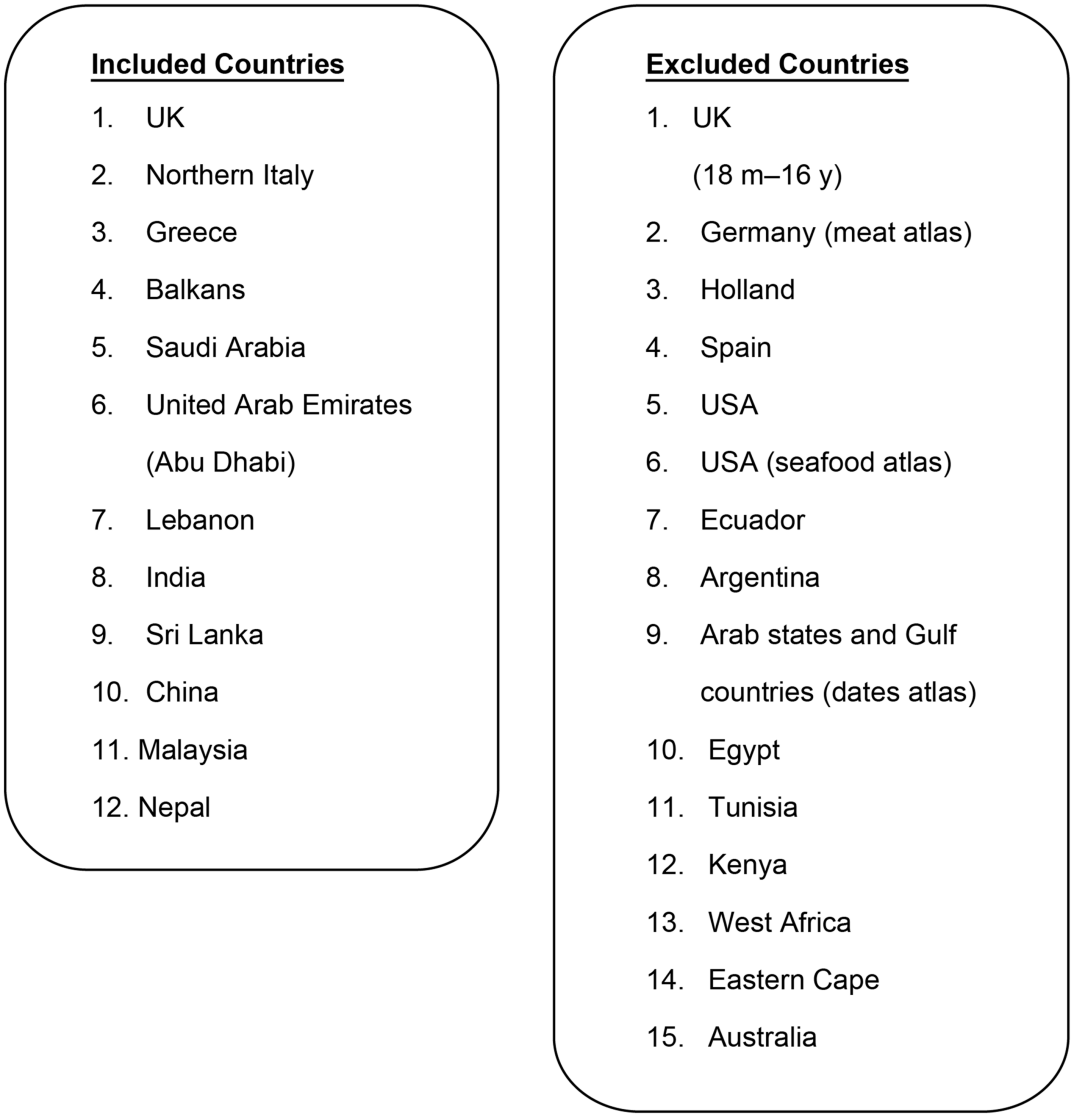
Included and excluded countries with food atlases. UK, United Kingdom; USA, United States of America.

The literature was included if it was specifically focused on food atlases, while studies that centered on other types of atlases, such as those related to economic complexity, human anatomy, or heart disease, were excluded from consideration. The extracted data (scientific literature) summarizes the countries with a food atlas; 27 countries were identified (10 from Asia, 8 from Europe, 5 from Africa, 3 from America, and 1 from Australia). A scoring system was developed to simplify decisions regarding the inclusion and exclusion of countries: nine domains (most popular food, photo, multiple portion size, clear photo, use representative cutlery healthy, adult (≥18 years), male, and female) were specified based on those included in the majority of food atlases; each of these nine domains was assigned one point; a score of 1 or 0 was given to each domain; if the total score was not equal to nine, the food atlas was excluded because each domain had to be represented in the food atlas framework included in this review (Table 1). Twelve and fifteen countries were included and excluded, respectively (Figure 1).

**Table 1:** Scoring system for the inclusion and exclusion of countries’ food atlases.

#	Country	Most popular food	Photo	Multiple portion size	Clear photo	Use representative cutlery	Healthy	Adult (≥18 years)	Male	Female	Score	Result
	Number of points	1	1	1	1	1	1	1	1	1	Total	
1.	UK	1	1	1	1	1	1	1	1	1	9	Ö
2.	UK (18 months–16 years)	1	1	1	1	1	1	0	1	1	8	X
3.	Germany (meat atlas)	0	0	0	0	0	0	0	0	0	0	X
4.	Northern Italy	1	1	1	1	1	1	1	1	1	9	Ö
5.	Holland	0	0	0	0	0	0	0	0	0	0	X
6.	Greece	1	1	1	1	1	1	1	1	1	9	Ö
7.	Spain	0	0	0	0	0	1	1	1	1	4	X
8.	Balkans	1	1	1	1	1	1	1	1	1	9	Ö
9.	USA	0	0	0	0	0	0	0	0	0	0	X
10.	USA (seafood atlas)	1	1	1	1	0	1	1	1	1	8	X
11.	Ecuador	0	1	1	1	1	1	1	1	1	8	X
12.	Argentina	0	0	1	0	0	1	1	1	1	5	X
13.	Arab states and Gulf countries (dates atlas)	0	0	0	0	0	1	1	1	1	4	X
14.	Saudi Arabia	1	1	1	1	1	1	1	1	1	9	Ö
15.	United Arab Emirates (Abu Dhabi)	1	1	1	1	1	1	1	1	1	9	Ö
16.	Lebanon	1	1	1	1	1	1	1	1	1	9	Ö
17.	Egypt	0	0	0	0	0	0	0	0	0	0	X
18.	Tunisia	0	1	1	1	1	1	1	1	1	8	X
19.	India	1	1	1	1	1	1	1	1	1	9	Ö
20.	Sri Lanka	1	1	1	1	1	1	1	1	1	9	Ö
21.	China	1	1	1	1	1	1	1	1	1	9	Ö
22.	Malaysian	1	1	1	1	1	1	1	1	1	9	Ö
23.	Nepal	1	1	1	1	1	1	1	1	1	9	Ö
24.	Kenya	1	1	1	1	1	1	0	1	1	8	X
25.	West Africa	0	0	0	0	0	0	0	0	0	0	X
26.	Eastern Cape province	0	1	1	1	1	1	0	1	1	7	X
27.	Australia	0	1	1	1	1	1	1	1	1	8	X

Ö, included; X, excluded; UK, United Kingdom; USA, United States of America.

## 3 Results

### 3.1 Overall description of a food atlas

A food atlas is an effective tool that relies on precise food consumption data to guide national and international health and nutrition policies (15). It comprises a collection of images depicting different amounts of food that has been widely studied and recognized as a valuable resource in dietary data collection, as well as for improving the accuracy of food quantification during dietary records and interviews. The accuracy of dietary assessments is enhanced by allowing users to select images from the food atlas that best represent the actual or typical size of the food portions they consume (16–18).

Most food atlases follow similar guidelines, including the photographic development standards, types of food items, number of portion sizes for each food item, and common household utensils and cutlery used as reference measurements. However, the content of food atlases varies depending on the country and its objectives. For example, Greece developed a food atlas to help assess the dietary intake in its population, whereas the UK used it to evaluate the quantity of food consumed by its population. The United States of America (USA) developed an atlas to understand the factors that influence food selection; Australia aimed to differentiate portion sizes through images; China sought to provide a visual reference to improve the accuracy of dietary surveys; Sri Lanka aimed to identify food portion sizes and varieties; and Southern Nepal used it to create and approve a photographic guide for dietary evaluation (19, 20). Food atlases are valuable tools in various countries for different purposes, and their use is expected to continue to grow in the future.

Many countries have adopted ideas from food atlases created in other nations, which vary based on factors such as the target audience, population, and gender. For instance, the Kenyan food atlas focuses on adolescents aged 9–14 years, while the Eastern Cape Province food atlas is designed for children aged ≤24 months. The UK Young Person’s food atlas is a collection of photographs used to evaluate the dietary intakes of children aged 18 months to 16 years. Food atlases can be created for specific health-related reasons and are considered reliable tools for medical professionals. For example, it could be challenging to explain a food atlas for treating dysphagia—a condition that requires texture-modified foods and thickened liquids—to patients and their caregivers. Additionally, some food atlases have been developed for particular types of food, such as the USA sea-food atlas, which measures seafood intake and its safety; the Gulf countries’ and Egyptian date atlases, which provide information on dates farmed in the Gulf Region; and the German Meat atlas, that supports climate justice and food sovereignty, and raises awareness of the environmental challenges posed by industrial meat production. These atlases present new information and facts and establish connections among various vital issues (10, 20, 21).

A photographic food atlas is a collection of images depicting different portions of various dishes that helps to estimate portion sizes. Various countries have created food atlases that represent the most consumed foods in society. The first food atlas was developed in Greece in 1992 and included approximately 170 food items. Food atlases have continued to develop worldwide until 2020. Table 2 provides a timeline of the countries that have developed food atlases, and the number of items included in these atlases.

**Table 2:** Timeline and number of food items of food atlases worldwide by country.

Year	Country (number of food items)
1992	Greece (170)
1994	UK (76)
2000	Argentina (118)
2005	Northern Italy (434)
2010	Australia (200)
2011	USA (N/A)
2012	Lebanon (212)
2013	India (247)
2014	UAE (Abu Dhabi) (83)
	Nepal (40)
	Germany (N/A)
2015	Holland (N/A)
2016	USA (seafood) (2)
	Eastern Cape province (N/A)
	Tunisia (N/A)
2017	Sri Lanka (125)
	UK (18 months–16 years) (104)
2018	Kenya (173)
	Balkans (135)
	Ecuador (68)
	Arab states and Gulf countries (60)
	West Africa (N/A)
	Saudi Arabia (231)
2019	Egypt (72)
	Spain (N/A)
2020	Malaysia (393)
	China (303)

UK, United Kingdom; USA, United States of America.

Most food atlases included 10 significant criteria that were determined after a review of global food atlases. As shown in Table 3, most countries agreed to meet most of the criteria: 96.3% of countries focused on food regarding the type of atlas, 74% of countries included multiple portion sizes in the atlas, and 70.4% included representative photos. The atlas target group was the public aged ≥18 years. By contrast, 11% of the food atlases focused on a target group or specific age group and represented specific food items, such as meat and dates.

**Table 3:** General food atlas criteria.

Criteria	Country	Number of country (*N* = 27)	%
Foods in the atlas represent the most consumed foods in the population	UK/UK (18 months–16 years)/Northern Italy/Greece/Balkans/USA/Ecuador/SA/UAE/Lebanon/India/Sri Lanka/China/Malaysia/Nepal/Kenya	15	55.6%
The atlas includes representative photos	UK/UK (18 months–16 years)/Northern Italy/Greece/Balkans/USA/Ecuador/SA/UAE/Lebanon/Tunisia/India/Sri Lanka/China/Malaysia/Nepal/Kenya/Eastern Cape province/Australia	19	70.4%
There are details regarding photography of the food in the atlas	UK/Northern Italy/Greece/Balkans/USA/Ecuador/SA/UAE/Lebanon/Tunisia/India/Sri Lanka/China/Malaysia/Nepal/Kenya/Eastern Cape province/Australia	18	66.7%
The atlas includes multiple portion sizes	UK/UK (18 months–16 years)/Northern Italy/Greece/Balkans/USA/Ecuador/Argentina/SA/UAE/Lebanon/Tunisia/India/Sri Lanka/China/Malaysia/Nepal/Kenya/Eastern Cape province/Australia	20	74%
The target group of the atlas is the public (adults aged ≥18 years)	UK/Northern Italy/Greece/Spain/Balkans/USA/Ecuador/Argentina/Arab states and Gulf countries (dates atlas)/SA/UAE/Lebanon/Tunisia/India/Sri Lanka/China/Malaysia/Nepal/Australia	19	70.4%
The target group of the atlas is a specific age group	UK (18 months–16 years)/Kenya/Eastern Cape	3	11%
The atlas is for food	UK/UK (18 months–16 years)/Germany (meat atlas)/Northern Italy/Holland/Greece/Spain/Balkans/USA/USA (seafood atlas)/Ecuador/Argentina/Arab states and Gulf countries (dates atlas)/SA/UAE/Lebanon/Tunisia/India/Sri Lanka/China/Malaysia/Nepal/Kenya/West Africa/Eastern Cape province/Australia	26	96.3%
The atlas is for items other than food	Egypt	1	3.7%
The atlas represents the most consumed foods by the society	UK/UK (18 months–16 years)/Northern Italy/Greece/Balkans/USA/Ecuador/SA/UAE/Lebanon/India/Sri Lanka/China/Malaysia/Nepal/Kenya	15	55.6%
The atlas represents specific food items, such as meat, dates, etc.	Germany (meat atlas)/USA (seafood)/Arab states and Gulf countries (dates atlas)	3	11%

UAE, United Arab Emirates; UK, United Kingdom; USA, United States of America.

### 3.2 Food items

The number of food items included in an atlas is not fixed; each country determines the number based on its needs or the information collected. The number of food items in each food atlas can vary for various reasons, such as determining commonly consumed foods based on the traditional diet of the community or selecting popular meals and dishes based on previous studies on food consumption in the region (1). For instance, Northern Italy had the highest number of food items (*n* = 434), followed by Malaysia (*n* = 393) and China (*n* = 303) (3, 18, 21). By contrast, Nepal had the lowest number of food items (*n* = 40), followed by the UK (*n* = 104) and the United Arab Emirates (UAE) (*n* = 115) (5, 19, 22). However, SA did not specify the number of food items included, while Egypt only collected 72 food items owing to the specialization of dates. Ecuador had difficulties estimating the nutrients in sauces and only collected 68 food items, while Nepal collected 40 food items due to the incorporation of more than one food item while eating; data were collected during only one meal. Half of the reviewed countries—including the UK (18 months–16 years), Balkans, Greece, Argentina, Lebanon, and Sri Lanka—averaged 138 food items (1, 2, 6, 10, 11, 23).

### 3.3 Collection methods

Different countries have various strategies to obtain information on the most consumed foods for inclusion in a food atlas. According to the data in Table 4, most countries used dietary or nutritional surveys, followed by FFQs, 24-h dietary recalls, and food recipe books (1, 8, 10, 11).

**Table 4:** Methods of identifying the most consumed food in countries worldwide.

Methods for included countries	Countries	Number of frequencies
Food frequency questionnaire	Greece/Argentina	2
The dietary or nutritional survey	UK/Balkans/Sri Lanka/Malaysia	4
24-hour dietary recall	Lebanon/Nepal	2
Data from previous studies	UAE	1
Food and nutrition websites	India	1
Questionnaire	SA	1
Recipe books	Northern Italy/India	2
Restaurant menu	Northern Italy	1
Referring to previous food atlas	Malaysia	1
Not determined	China	1

Other countries collected food consumption data using means such as FFQs, 24-h dietary recalls, pilot studies, food-related databases, cookbooks, and restaurant menus. For instance, the UK food atlas for children relied on the dietary and nutritional surveys of children aged 1.5–4.5 years, young people aged 4–18 years, and a pilot study, resulting in a total of 104 food items. Similarly, the food atlas of Australia includes food items from the “Diet Advice” website database, which were individually assessed to determine the number of foods requiring portion images for the accurate reporting of dietary intake (24).

The photographic food atlas of Kenyan adolescents in Nairobi County uses data from a variety of families with low-to-middle income backgrounds to represent the foods most consumed by Kenyan adolescents (25). By contrast, the Seafood Atlas of the USA was based on pilot studies demonstrating that tilapia filets and white shrimp were suitable for producing generic fish and shrimp photographs (21). In Sri Lanka, various methods have been used to gather information on food consumption, including data from a nutritional survey and the development of an FFQ for Sri Lankan adults.

In Sri Lanka, the urban population’s increased consumption of Western foods has led to their inclusion in the food atlas, with an input from several nutritional experts who believe that they might be essential for future use (11). For the Balkan region, the food atlas selection was based on prior food consumption surveys. Traditional cookbooks and restaurant menus were consulted to include additional dishes that reflect the local dietary patterns and cultural competency (1). In the UAE, the most consumed foods were determined using previous food consumption data, food atlases, and food composition tables, as well as data collected from Gulf Cooperation Council countries and the Middle East. This includes traditional UAE and Middle Eastern foods, which are characterized by distinct ingredient compositions (26). The Tunisian food atlas relies on epidemiological studies published between 1996 and 2005 to assess a wide range of food items and portions (27).

Different countries have employed various methods to select foods for the atlas based on their capabilities. For instance, Malaysia referred to previous food atlases, related documents, current national food consumption data, and researchers’ observations of readily available market foods (22); India searched food and nutrition websites; and Northern Italy sought assistance from Italian diet recipe books, restaurants, cafeteria menus, and the most consumed dishes (3). In the UAE, data were extracted from previous studies, SA used a questionnaire, and China has yet to determine an exact method of collection (12, 26, 28).

### 3.4 Utensils, photography, and portion sizes in food atlases

**Figure 2:**
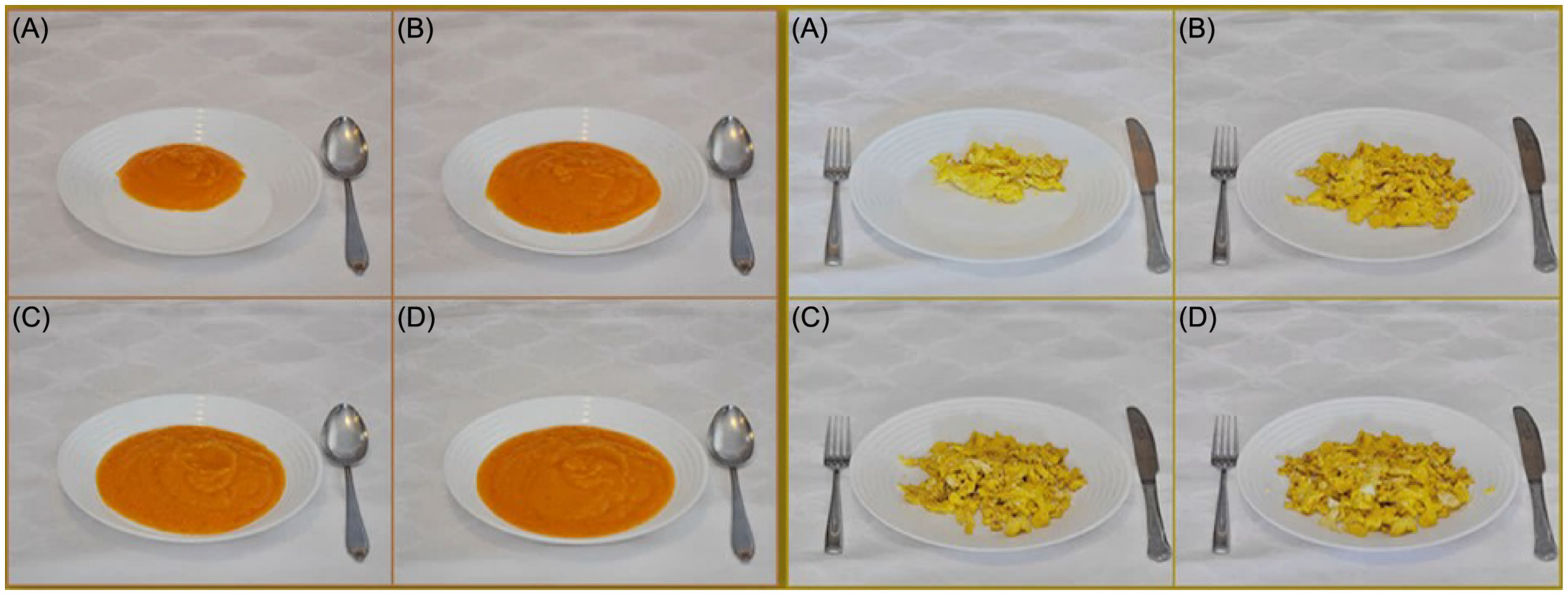
Utensils in the Balkan Region food atlas [Photo series of carrot soup (Left **(A—D)**)] and scrambled eggs [right **(A—D)**].

The selection of traditional and commonly used utensils that represent a community’s eating habits and behaviors plays a critical role in determining consumption and enhancing the accuracy of using the food atlas as an assessment tool. The most used utensils—such as plates, bowls, and cups in countries like the UK, UAE, Italy, Greece, Balkan, Lebanon, Tunisia, Sri Lanka, and Australia—are standard white utensils used to present mostly consumed food items (Figure 2).

**Figure 3:**
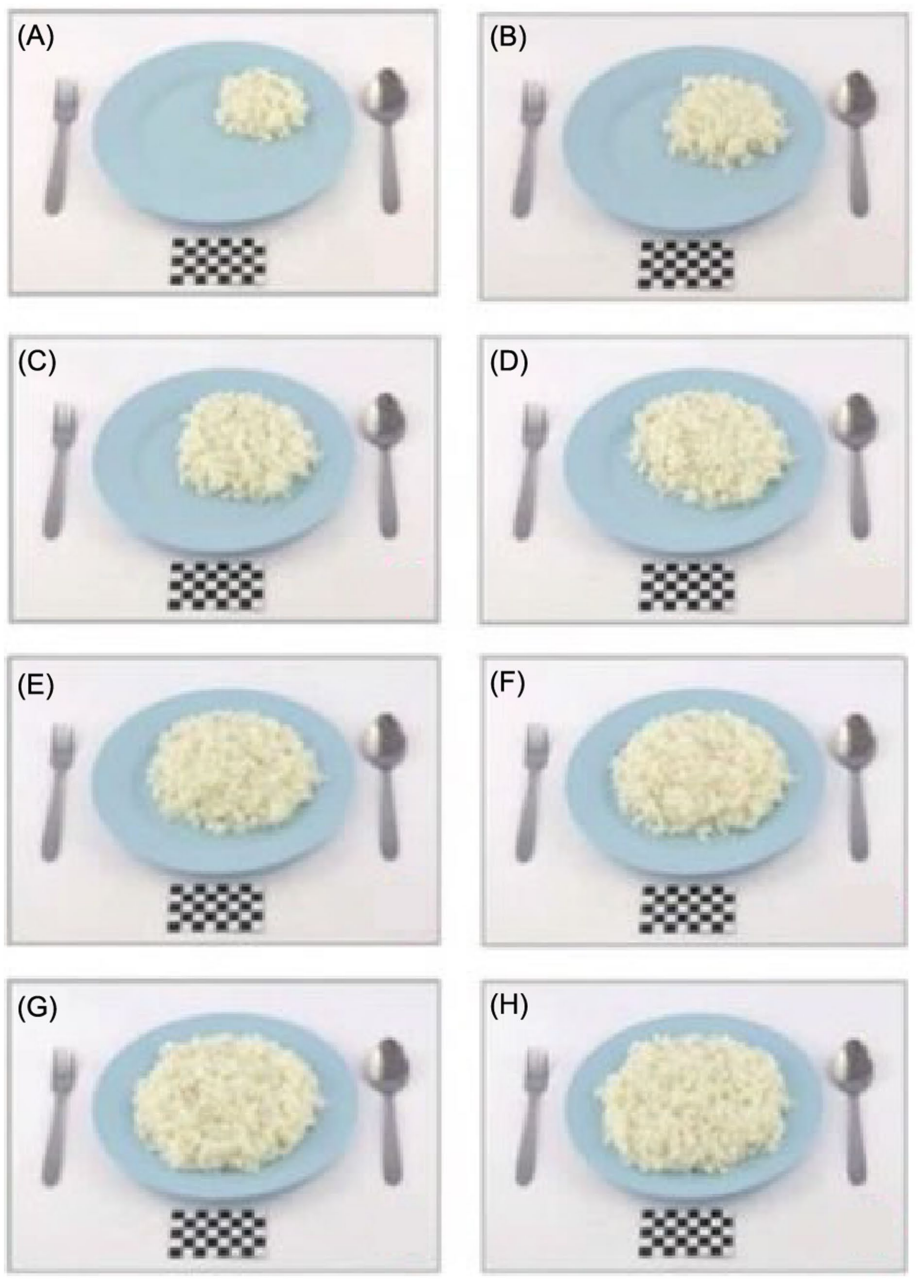
Utensils in the Malaysian food atlas [Photo series of rice **(A—H)**].

By contrast, the utensils used in the Ecuadorian atlas are light brown plates with six sets of white measuring cups to represent their culture, whereas Malaysia used a plain baby-blue plate to represent food items (Figure 3).

**Figure 4:**
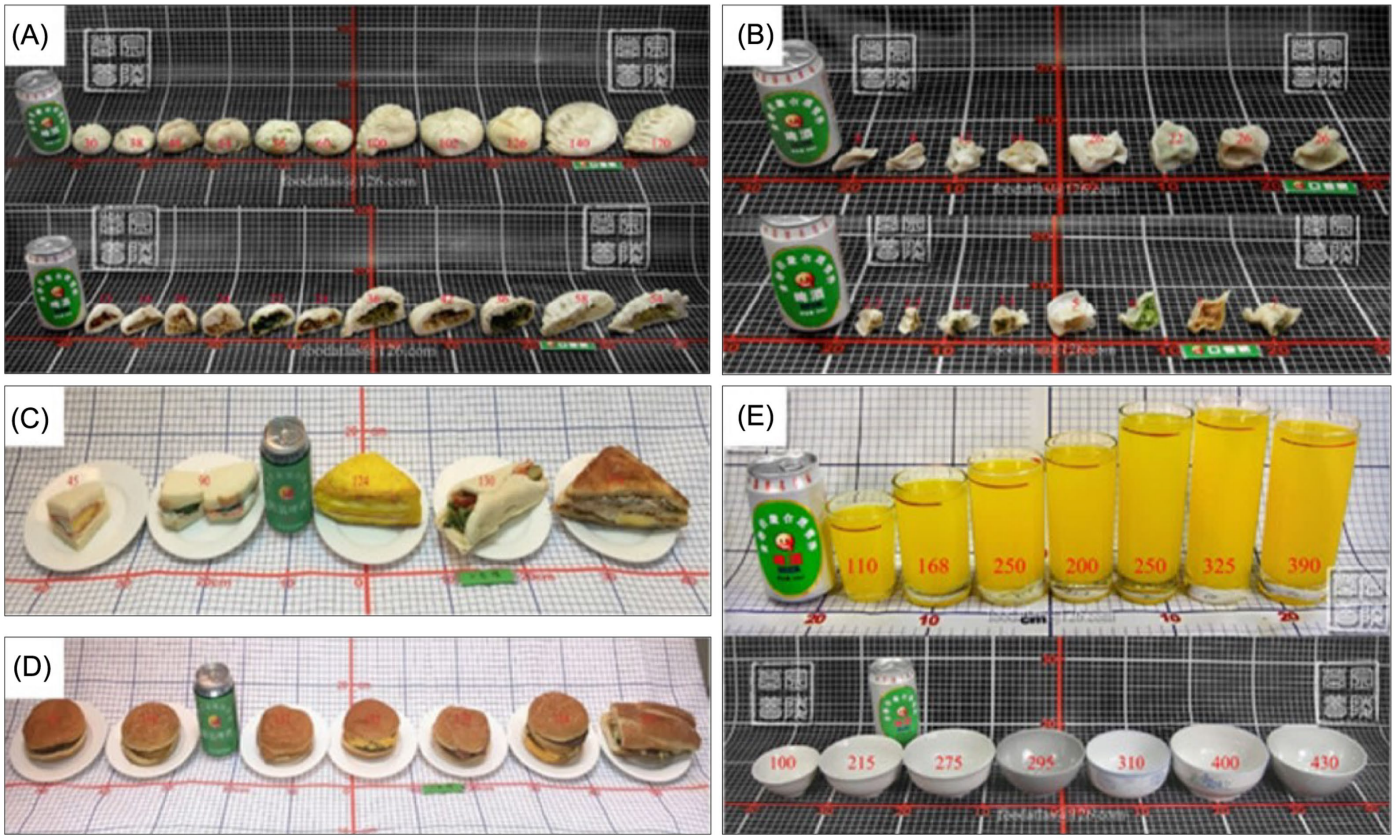
Utensils in the Chinese food atlas (Photos of Chinese and Western representative compound processed foods and tableware. **(A)** Steamed stuffed buns. **(B)** Dumplings. **(C)** Sandwiches. **(D)** Hamburgers. **(E)** Glasses (above) and bowls (below) of different sizes).

Furthermore, countries such as China choose utensils based on the color, shape, and amount of food; thus, plates of different colors and sizes were selected (Figure 4).

**Figure 5:**
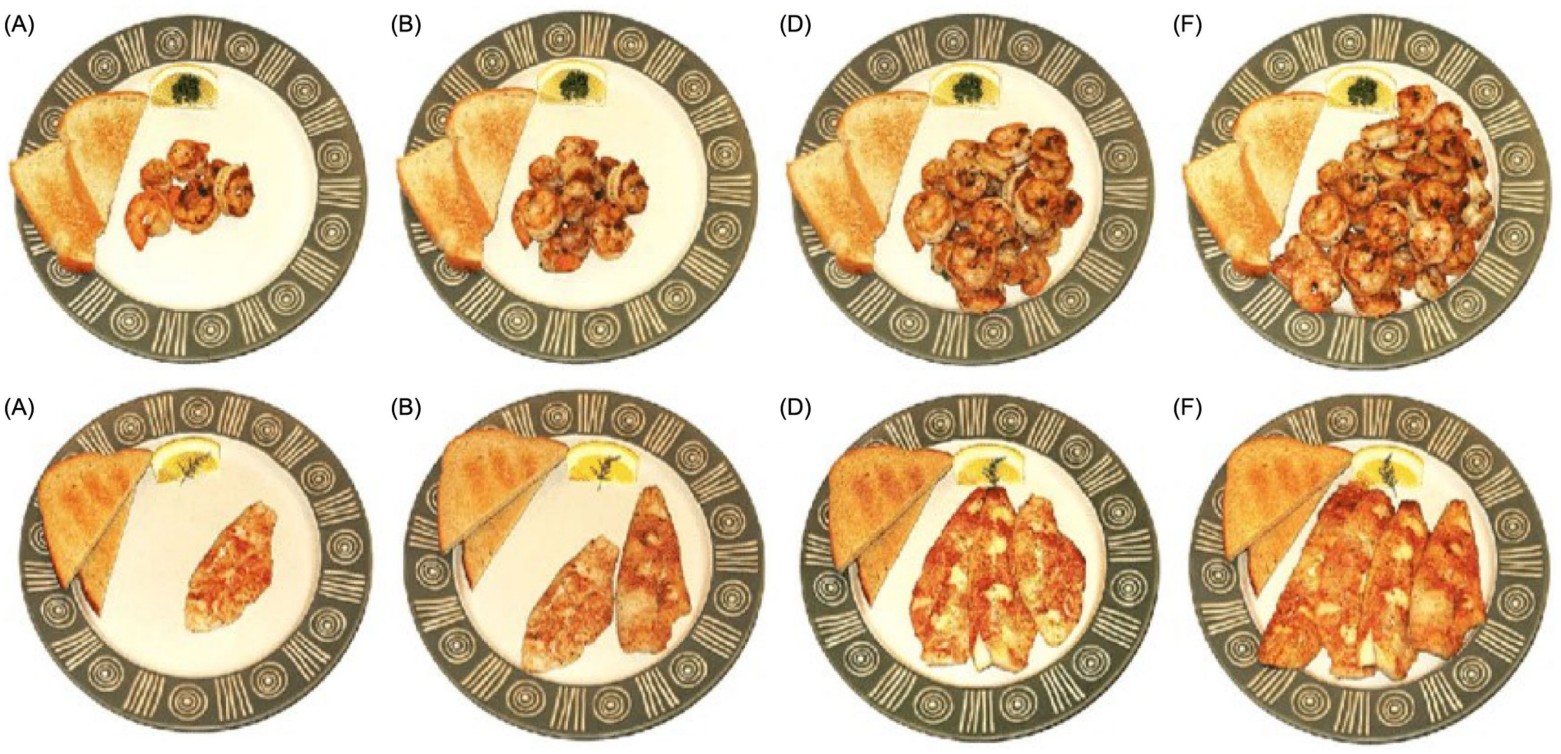
Utensils of the USA Seafood atlas [Photo series of shrimp and fish portion **(A—F)**].

Generally, the variations in the utensils selected in a food atlas can be attributed to the traditional and cultural differences in the eating habits and behaviors in each country. To help assess and estimate the actual amount or portion of food consumed by users of the food atlas, most of the reviewed food atlases place spoons, forks, and knives next to the plates. For instance, the US food atlas places a piece of toast and garnished lemon wedge on the same seafood ceramic plate as a reference object (Figure 5).

The quality of the photographs is crucial for determining the appropriate portion size to represent the quantity of food consumed, and clear information about the cameras and tools used to photograph the food is necessary. In the Balkan food atlas, a professional photographer was hired to capture photographs of food portions under standard lighting conditions on a white background (1). The same conditions were applied in all photographs, the weight of the food portion was marked on the photograph, and images were presented in color to make them appear realistic. Different methods have been used to determine the portion sizes of food items in food atlases. In Greece, a questionnaire was administered to participants to report their usual portion sizes, and the 5th and 95th percentiles of the reported intake were selected to correspond to the quantities presented in the first and last pictures in each photo series of the atlas. Equal increments on a log scale were used to estimate the amount presented in the intermediate images (2). In Australia, the “Food Works” nutrition analysis software was used to determine the average size of portion sizes to portray a graduated increase in portion size (24). In the UAE, the weights for each portion size were obtained directly from the UK food atlas, and a household survey of local families was conducted to provide the required data on the portion sizes of traditional foods not stated in the UK food atlas (26).

Most food atlases used a series of photographs with food of different weights and sizes to provide multiple choices for common or nutritionally essential food items, such as rice, to determine portion sizes (2, 6, 24, 26). By contrast, Lebanon and Tunisia only used three portions for each food item (10, 27); we assumed that the portion sizes consumed were representative (27). Conversely, China designed 4–10 grades of food portions based on food size, quantity, or a set number of portions within the range of the most consumed amount (19). Generally, the weights of food portions were directly measured using electronic scales to the nearest gram (or milliliter for liquids) (19). Estimating portion sizes has been a significant limitation of dietary studies in the past, since estimates can be less accurate than weighed portions (6, 21). However, printed photographs of foods have shown increased accuracy in food portion estimation compared with unassisted estimates (6, 21).

## 4 Discussion

Food atlases assist in determining and estimating nutritional intake and are used in nutritional surveys, assessments of patients, evaluations of food consumed by populations, assessments of dietary habits and behaviors of society, and their relationship to weight gain. It also helps assess food consumption by age group and is used in FFQs.

In most countries, food atlases rely on national surveys and questionnaires to identify commonly consumed food items. However, some countries use a combination of methods to cover all types of consumed food items. Owing to differences in the methods used for identifying the most consumed foods in each country’s food atlas, there is no consumption information methodology that can be considered the gold standard. The choice of a method depends on factors such as available resources, data, cost, time, and suitability for the developing team and population.

Food atlases contain photographs of food items describing traditional household utensils and portions. Ensuring the quality of food photographs thus requires consideration of all elements, such as lighting, camera, position, distance, angle, reference objects, and background. Many factors, including personal and food characteristics, affect the estimation of portion size by individuals and may lead to significant estimation errors. Therefore, determining the amount of food consumed by individuals remains a challenge in accurately estimating food portion sizes.

**Figure 6:**
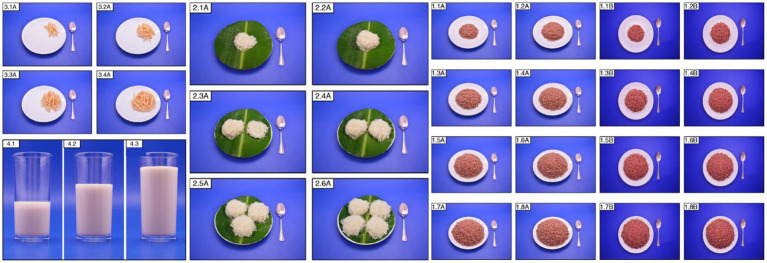
Photos in the Sri Lankan food atlas [Photo series of french fries (3.1A-3.4A)], milk (4.1-4.3), string hoppers (2.1A-2.6A), and cooked red rice (1.1A-1.8B).

Despite these challenges, the development of a national food atlas offers several advantages. For instance, conducting studies to identify traditional dishes to be included in the atlas, such as in the Lebanese food atlas; and using accurate estimation and quantification for each food, along with color photos to attract participants, such as in the Sri Lankan food atlas (Figure 6).

### 4.1 Future steps in creating food atlases

Following a comprehensive review of food atlases globally, there has been a call for a scientifically unified guide to develop national food atlases. In response, a procedural manual was created to outline the steps required to create a food atlas that accurately reflects a community’s food consumption. The manual was developed based on a thorough examination of relevant studies.

**Figure 7:**
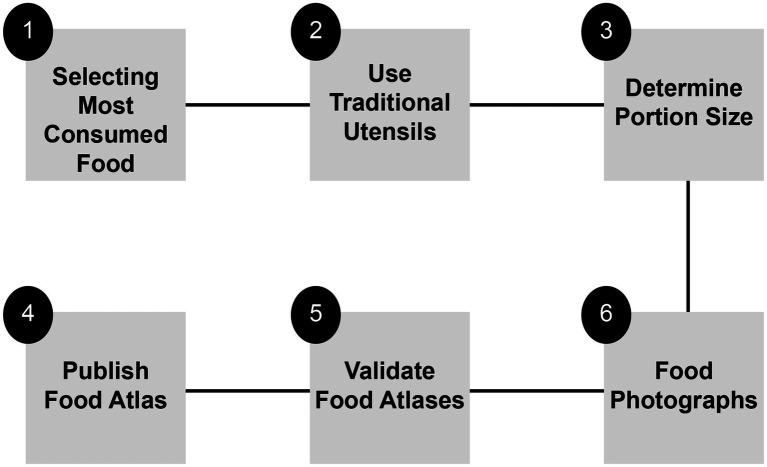
Process of developing a food atlas.

A steering committee was established to ensure the accuracy and scientific rigor of the food atlas. The committee—comprising experts from various sectors (including government and private organizations) and possessing professional expertise in food, nutrition, education, psychology, public health, and other related fields—was responsible for providing scientific recommendations and conducting a thorough review of the food atlas outputs. The development of a food atlas involves six distinct stages (as illustrated in Figure 7).

First, selecting the most consumed food is a critical step in creating a food atlas. The foods chosen for the atlas should accurately represent a country’s food consumption patterns. There are various methods for identifying typical foods in a specific region, and the choice of the most appropriate method should be based on the availability of data and resources (see Supplementary material for further details).

Second, selecting a representative utensil from a community. The public typically uses traditional utensils; thus, selecting a representative utensil plays an active role in the success of the food atlas. While determining traditional and commonly used utensils may require efforts, it will be helpful to improve the accuracy of the food atlas assessment (see further details in Supplementary material).

Third, portion size must be determined. The food portion size is determined based on the typical food intake of the population. Selecting the average portion size as a representative method for food consumed by individuals in the community is crucial (see Supplementary material for further details).

Fourth, photographs of the food are captured. Minimizing errors in portion size estimates is one of the most significant challenges in creating and photographing food atlases. These errors are influenced by the interaction between the format of the photograph series and the participants’ skills in describing the portion size. The primary factors that are most likely to affect the interaction include the size of the images, portion sizes, interval, order of presentation, labels, background, color, and camera type. Although the protocols varied among different countries, the most common protocol was selected for the procedural manual (see Supplementary material for further details).

The fifth step is the process of validating food atlases, which is crucial because it confirms the accuracy and reliability of the atlas for use. Two methods were used to validate the atlas: visual estimation of the quantity of food, and recall information using the atlas (see Supplementary material for further details).

Finally, once validated, the food atlas is ready for publication. Technology plays an important role in ensuring success; thus, the atlas is published in multiple versions based on community preferences. Moreover, the availability of both digital and paper versions is essential for reaching people of all ages in society (see Supplementary material for further information).

The development of food atlases worldwide has identified certain disadvantages that must be avoided when creating a nationally representative food atlas. One of the disadvantages is the difficulty in determining the appropriate portion size and mixing different types of food; for example, the addition of cooked beans to white rice may affect dish density, leading to inaccurate portion-size estimation and photography. Additionally, variations in food recipes across different regions within the same country can result in differences in nutritional factors, such as calorie intake. Furthermore, limitations in the timing of food selection, such as only during lunchtime, can result in a lack of representation of dishes consumed at different times of the day. The use of the Scopus database for literature search presents another limitation, because it is crucial to extend the range of research metrics to encompass nearly twice the number of peer-reviewed publications. However, as a reputable source of information, the Scopus database is widely recognized for its extensive global data coverage.

Several potential strategies may address challenges related to food atlases, including accounting for regional variations and culturally sensitive portion sizes across different areas of Saudi Arabia. Additionally, emphasis should be placed on using consistent methodological approaches when determining appropriate portion sizes and recognizing variations in food recipes throughout the region. Collaboration across disciplines is also essential. By adopting these strategies, food atlases are expected to become more inclusive, accurate, and applicable to diverse cultural and methodological contexts.

## 5 Conclusion

Food atlases play a crucial role in promoting and optimizing nutrition and food-related aspects worldwide by providing a unified scientific method for their development. The aim of creating a food atlas is to establish an authoritative reference for the quantitative evaluation of food intake by community members, while reducing the likelihood of errors during the assessment process. This study emphasizes the importance of food atlases and provides a six-step procedure manual that can be used as a reference for their development. Further research is necessary to confirm the efficacy of the proposed manual, to streamline and standardize national initiatives. Moreover, given the widespread digital transformation currently occurring both globally and within individual countries, it is essential to conduct future studies that assess the feasibility of digitizing food atlases, thereby reducing the time required for their completion.
