# Supplementary material for: Comprehensive global review and methodological framework for developing food atlases
Source: Front Nutr. 2024 Nov 22;11:1505606. doi: 10.3389/fnut.2024.1505606 (PMC11620897; doi:10.3389/fnut.2024.1505606)

Supplementary Material

Procedure Manual for Developing Food Atlases

<u>2021</u>

Table of Contents

DEFINITIONS..4

SCOPE..4

TARGET POPULATION..4

INTRODUCTION..5

FOOD ATLAS..5

FOOD ATLASES DEVELOPMENT PROCESS..5

THE TEAM..7

PROCESS OF DEVELOPING FOOD ATLAS..9

STEP ONE: SELECTING MOST CONSUMED FOOD..10

STEP TWO: USE TRADITIONAL UTENSILS COMMONLY USED BY COMMUNITY MEMBERS. 10

STEP THREE: DETERMINE THE PORTION SIZE. 10

STEP FOUR: FOOD PHOTOGRAPHS..10

STEP FIVE: VALIDATION OF FOOD ATLASES..10

STEP SIX: FOOD ATLAS PUBLICATION..10

REVERENCES.. **ERROR! BOOKMARK NOT DEFINED.**

Definitions

Name	Indication
**The project**	Food atlas development
**Scientific team**	Principal Investigator and a number of associate researchers
**The Committee**	Steering Committee
**Outcomes**	Final results

Scope

This procedure manual describes the steps for developing a photographic food atlas.

Target population

This manual is aimed at organizations, authorities, researchers, and nutritionists who plan to develop food atlases.

Introduction

Proceeding from the vision of Saudi Arabia 2030, with one of its axes being based on a “a vibrant society to be the foundation for a prosperous economy and an ambitious nation,” aimed to contribute to activating and strengthening the role of the National Nutrition Committee (NNC) in Saudi Food and Drug Authority (SFDA) by providing its recommendations and scientific opinions in the field of nutrition to relevant parties to improve the nutritional and health Status in the Kingdom of Saudi Arabia to build a healthy society. The NNC discussed the need to develop a guide to clarify the actual consumption of food by community members in Saudi Arabia that will help:

- Determine and estimate the nutritional intake.
- In the nutritional survey.
- In the nutritional assessment of patients.
- In a quantitative assessment of the food consumed by the population.
- Assess the nutritional habits and behaviors of the Saudi society and their relationship to weight gain.
- Assess the amounts of food consumption by age group.
- In the food frequency questionnaire.

Accordingly, the general secretariat of NNC prepared this procedure manual to clarify how to develop a food atlas that represents the consumption of food by the community. The procedure manual is developed based on benchmarking study of food atlases that were carried out in twenty-two countries (regional and international).

Food atlas

A food atlas is a book that includes a wide range of pictures of foods in different amounts or sizes that are used to estimate portion sizes. An atlas usually contains:

- Food photography.
- Types of food items (dishes).
- Numbers of portion sizes for each food item.
- Numbers of commonly used household utensils and cutlery that are used as reference measurements.

The food atlas may differ from one country to another based on its contents. For example, there are atlases that specialize in:

- Determining the size of food portions (such as the atlas of the Balkans region).
- One type of food (such as the atlas of Germany that specialize in meat, and the atlas of Egypt that specialize in dates).
- Specific age groups (such as the atlas of the Eastern Cape Province that focuses on children, and the atlas of Kenya that focuses on teens).

A unification of scientific method for the development of a food atlas is needed, which in turn will be considered an approved reference that contributes to the quantitative assessment of food according to the actual consumption of food by community members, in addition to reducing potential errors that may occur during the assessment of food consumption.

Food Atlases Development Process

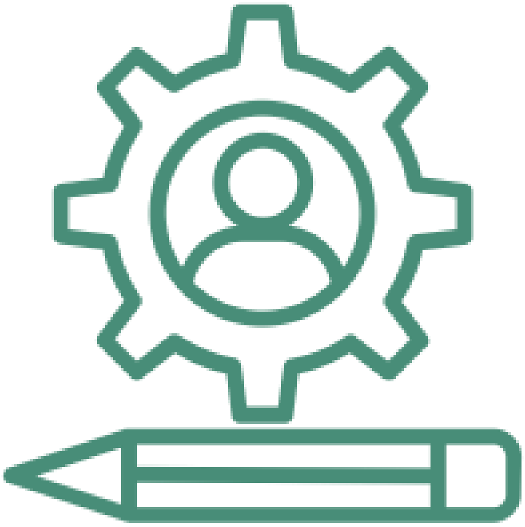

frontiers

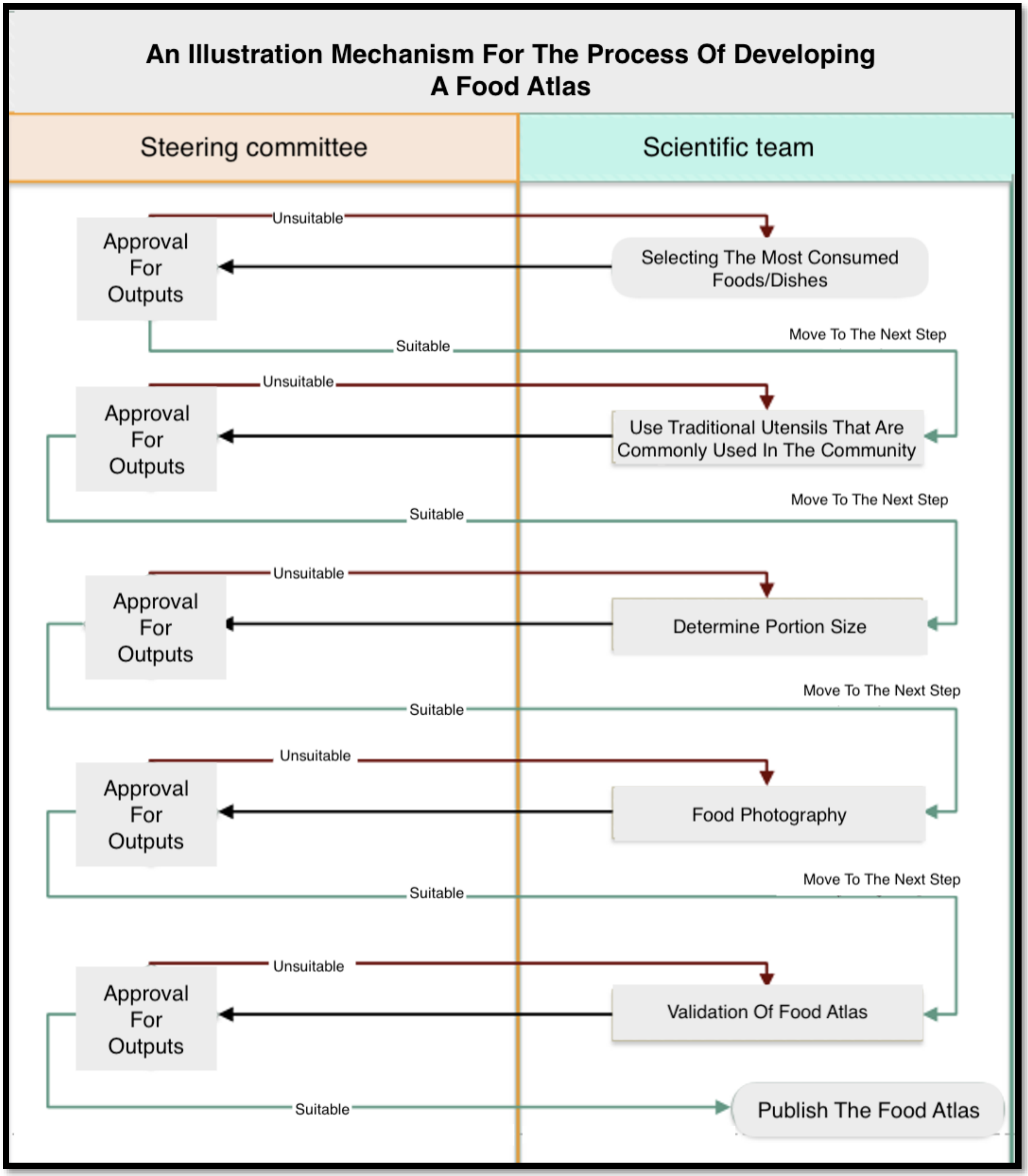

An Illustration Mechanism For The Process Of Developing A Food Atlas
Steering committee
Scientific team
Unsuitable
Approval For Outputs
Selecting The Most Consumed Foods/Dishes
Suitable
Move To The Next Step
Unsuitable
Approval For Outputs
Use Traditional Utensils That Are Commonly Used In The Community
Suitable
Move To The Next Step
Unsuitable
Approval For Outputs
Determine Portion Size
Suitable
Move To The Next Step
Unsuitable
Approval For Outputs
Food Photography
Suitable
Move To The Next Step
Unsuitable
Approval For Outputs
Validation Of Food Atlas
Suitable
Publish The Food Atlas

Scientific team

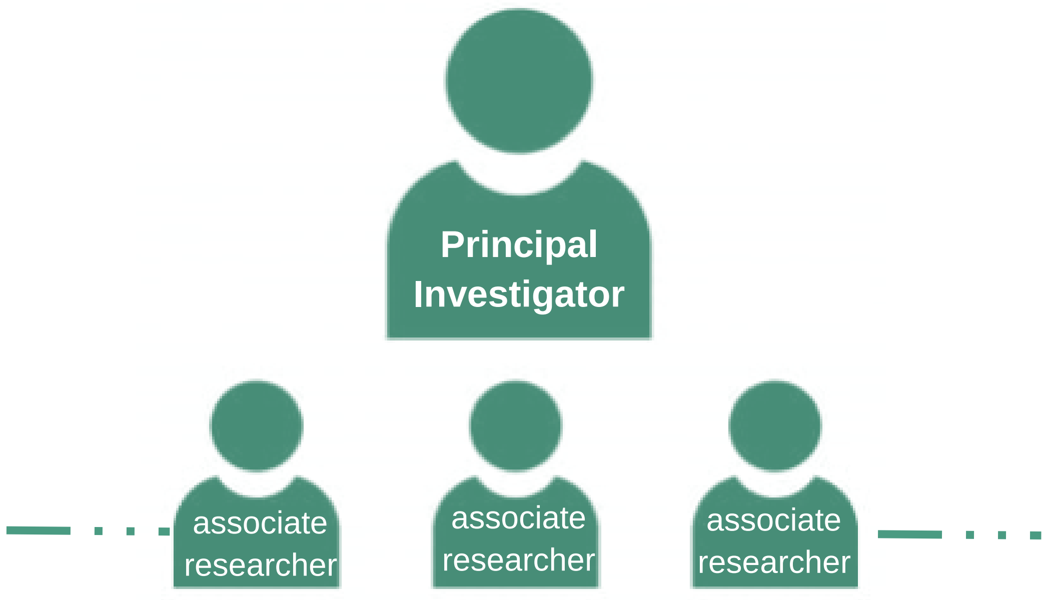

-The scientific team consists of the principal investigator and several assistant researchers.

-The principal investigator has absolute freedom to form the appropriate team to work on the project.

Tasks of the team

-The team follows the directions in the procedural guide to work on implementing the project and completing it to the fullest extent.

-The team works on the recommendations of the steering committee under the guidance of the principal investigator.

The Steering Committee

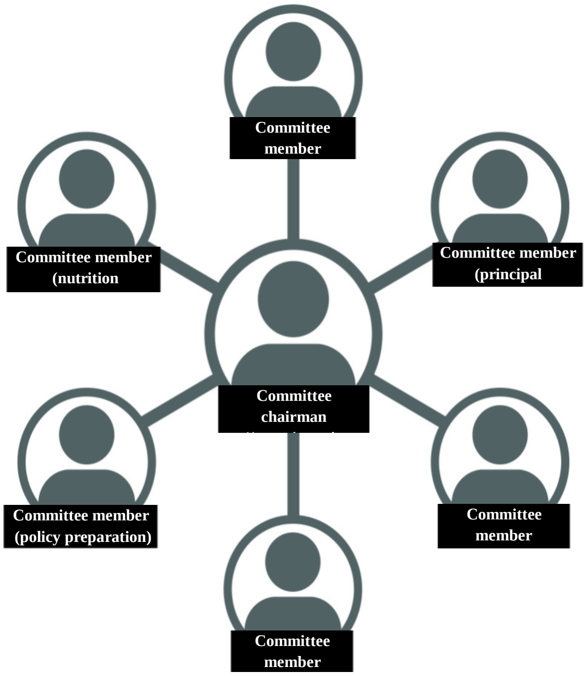

- The steering committee is responsible for reviewing the project outcomes.
- The steering committee is chaired by individuals with expertise in atlases.
- The committee should include the principal investigator as a member.
- Committee members must be different from the project team members (except for the principal investigator).
- The committee is composed of several members representing different specializations and entities (governmental and private) who have experience in food, nutrition, education, psychology, public health, or any experience related to food atlases.
- The committee should include experts in policies and programs related to food and nutrition.

Tasks of the Committee

- The steering committee communicates effectively with the scientific team through development of scientific recommendations and the accurate scientific review of the output.
- The steering committee approves the final scientific outputs from the scientific team for every step and at the end of each step before moving on to the next one.
- The steering committee must provide the scientific team with adequate levels of support (strategic, financial, and technical).
- The steering committee must ensure that there are adequate levels of communication and supervision of the review process within the scientific team.
- The chairman of the committee makes the final decision.

Steps of developing food atlas

Step One: Selecting Most Consumed Food

Step Two: Use Traditional Utensils Commonly Used by Community Members

Step Three: Determine the Portion Size

Step Four: Food Photographs

Step Five: Validation of Food Atlases

Step Six: Food Atlas Publication

Step One: Selecting Most Consumed Food

- Many food atlases have been developed around the world by different countries, and the stage of reviewing previous efforts (regionally and internationally) to develop food atlases and distinguishing them from each other is one of the most important stages that helps determine the appropriate standards for developing a food atlas that represents the actual consumption of food by members of society in any country.
- When developing a food atlas, one of the most important steps is choosing the foods that will be included in the atlas. The types of foods (dishes) chosen for the food atlas must be representative of the actual food consumed by individuals in the community that are then divided into food groups.
- There are many methods that are used to determine the most consumed foods (dishes), and in this guide several methods will be reviewed that were chosen based on the standard review of several food atlases.

<u>*Note: The work team can choose the best method from the seven methods below or combine the outputs of more than one method, based on the availability of data and resources.</u>

1

Selection from national nutrition survey

This method depends on the availability of surveys in the country:

A: If a survey is available:

Foods (dishes) can be selected based on nutritional surveys previously conducted in the country or region. This method is known to be the easiest, most documented, and most representative of the foods most consumed.

B: If a survey is not available:

A nutritional survey can be performed; however, this method may be difficult and expensive. Thus, another method can be chosen to select foods (dishes) in the atlas.

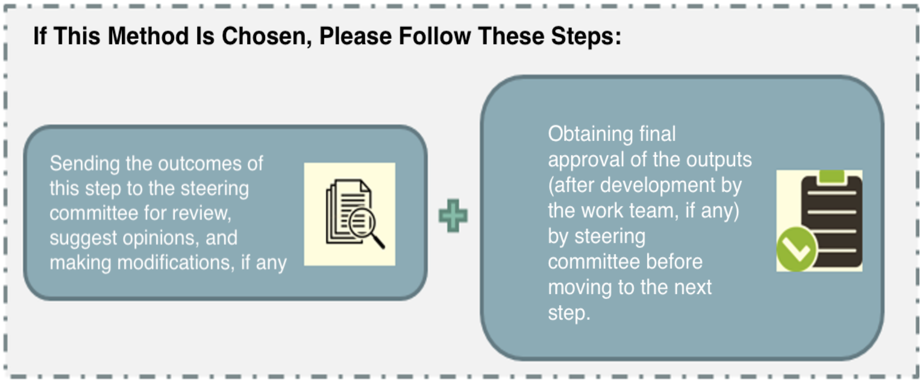

2

Food Frequency Questionnaire

This method depends on the availability of the questionnaire in the country.

A: If a food frequency questionnaire is available:

Use the questionnaire that was previously developed to measure food consumption in the country. This method is more valid and reliable.

B: If a food frequency questionnaire is not available:

*Note: The steps below are a summary of the most important points involved in developing an food frequency questionnaire. Please develop your food frequency questionnaire by referring to reliable scientific references and having it approved by the Steering Committee before initiation.

A new questionnaire can be developed to determine the most consumed foods (dishes) as follows:

1. Determine the target group for the questionnaire (age group, gender, and region).
2. Select samples through a multi-stage random sampling to ensure that the sample is representative of the population.
3. Select the foods (dishes) for the questionnaire using the methods mentioned below:
   - A. 24-Hour Dietary Recall over the past 24 hours for a representative sample
   - B. Weighing food for 3 days for a representative sample
   - C. Open-ended questions to find out details about eating fruits and seasonal foods on occasions such as holidays and Ramadan.
   - D. Foods (dishes) mentioned by local nutritionists.
4. Sort similar foods into different groups based on the nutritional content and suitability (such as grains, starches, vegetables, legumes, fruits, meats, fats, beverages, and desserts).

5. To improve the quality of the questionnaire, color photographs of foods (dishes) can be used to help estimate the serving size, especially for foods for which it is difficult to determine the serving size.

6. Conduct a reliable study with the aim of verifying the validity of the questionnaire (smart selection from the specified category and testing the evidence on it. At the same time a direct nutritional recall report is conducted within 24 hours (24-hour Dietary Recall) from the sample, and the results of the questionnaire are compared with the nutritional recall report daily within 24 hours (24-hour Dietary Recall).

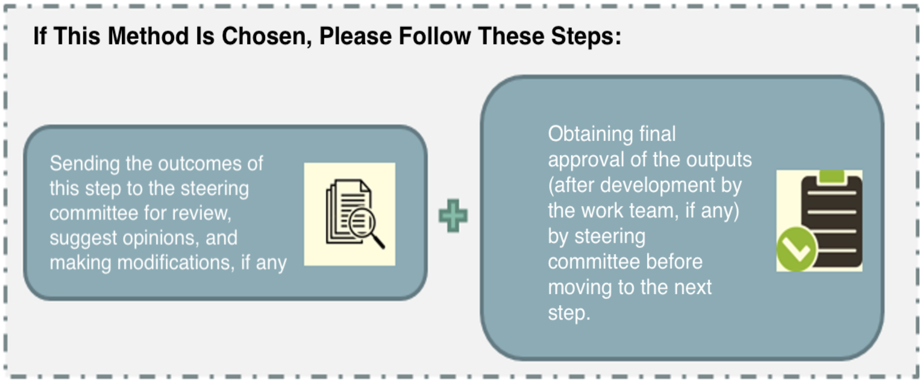

3

Choose from menus of restaurants and cafes.

This approach is appropriate to use in countries where people consume the most food from restaurants. Foods (dishes) are selected from restaurants as follows:

1. Select restaurants randomly from a list containing the names of all restaurants in the country to ensure it is a representative sample.
2. Review restaurant menus and choose the most consumed food from the menus.
3. Evaluate the validity of the data collected from restaurants through a survey of the target group to ensure the accuracy of the data.

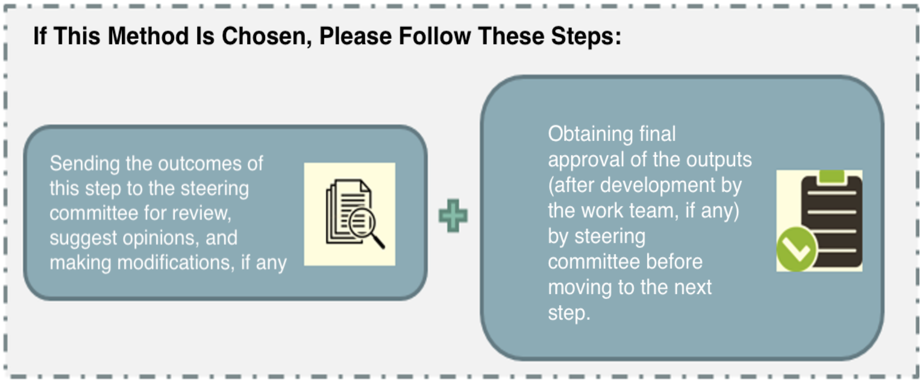

4

Choose from cookbooks, recipe websites, or social media sites.

Choose any of the three methods below or combine the outputs of more than one method.

1. Identify the most widely used cookbooks in the country as follows:
   A. Collect data from libraries (in-store or online library sites).
   B. Conduct a survey to find out the most frequently used cookbooks.
   C. Sort recipes from books and divide them into categories according to the food groups identified for the project.
   D. Evaluate the validity of data taken from cookbooks through a survey to ensure the accuracy of the data.
2. Identify the most widely used electronic prescription sites in the country as follows:
   A. Collect data from search engines regarding the most visited websites.
   B. Conduct a survey to find out the most visited websites by individuals.
   C. Sort recipes from websites and divide them into categories according to the food groups identified for the project.
   D. Evaluate the validity of data taken from websites through a survey to ensure the accuracy of the data.
3. Identify the most visited social media sites in the country as follows:
   A. Collect data from social media sites.
   B. Conduct a survey to find out the most visited social networking sites.
   C. Sort recipes from social media sites and divide them into categories according to the identified food groups.
   D. Evaluate the validity of data taken from social media sites through a survey to ensure the accuracy of the data.

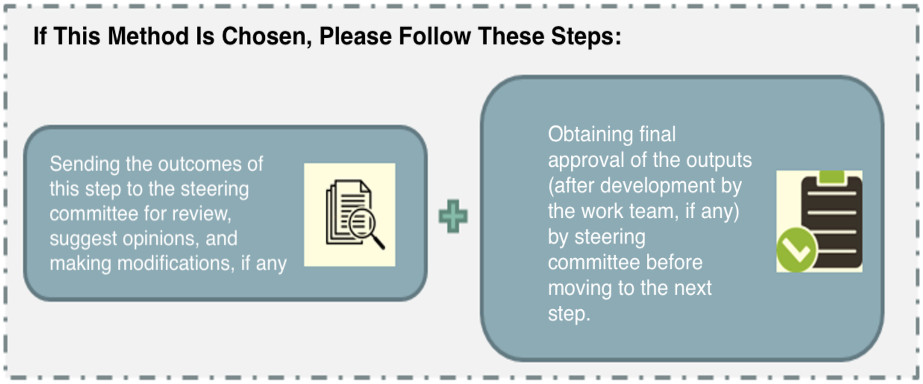
If This Method Is Chosen, Please Follow These Steps:
Sending the outcomes of this step to the steering committee for review, suggest opinions, and making modifications, if any
Obtaining final approval of the outputs (after development by the work team, if any) by steering committee before moving to the next step.

5

Selection of epidemiological studies

1. Review previous epidemiological studies that were conducted to determine the foods (dishes) most consumed in the country.
2. Collect and analyze data from the results of studies.

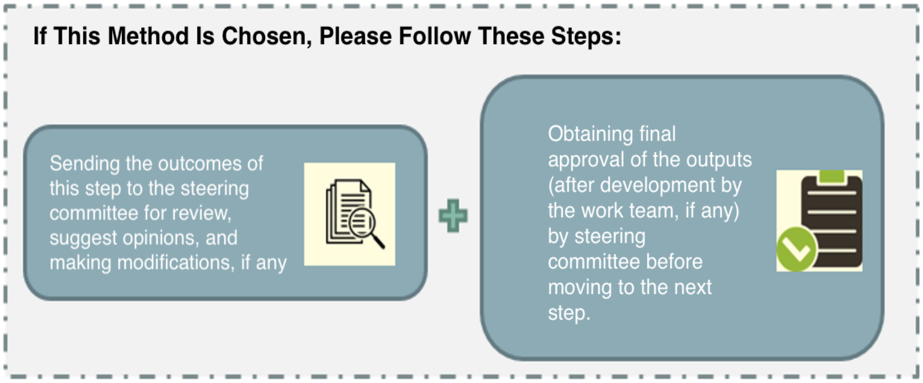

6

Choose from experimental studies or descriptive cross-sectional studies.

1. Conduct an experimental or a descriptive cross-sectional study aimed at identifying the most consumed foods.

*Note: The type of study is determined according to resources and what the staff deems appropriate.

2. Select the sample randomly according to the type of the study.
3. Use a Food Frequency Questionnaire, direct food recall over the past 24 hours (24-Hour Dietary Recall), a 3-day weight record, or interviews (depending on the type of the study) to collect data related to foods.

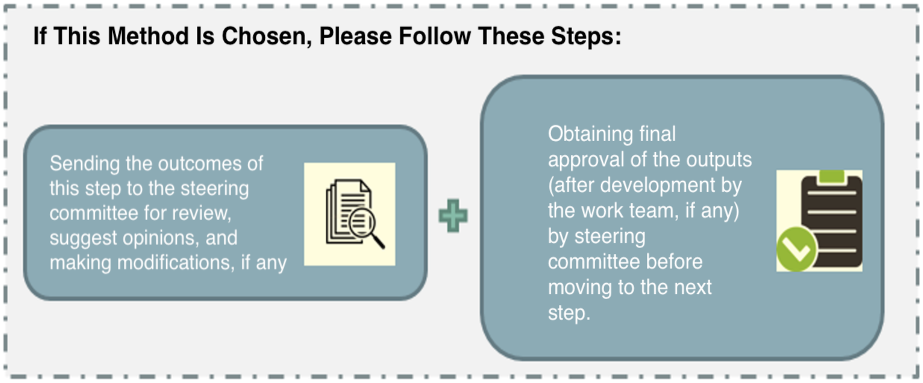

7

Choose from databases comprising data collected from the country.

Use food databases comprising data from the same region to determine the most consumed foods as follows:

1. Choose the most appropriate food database that represents the most consumed foods.
2. Determine the correct methodology for selecting foods from the database.

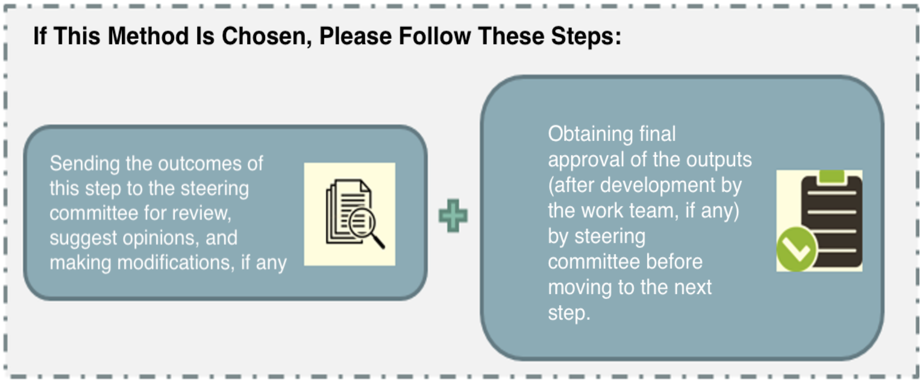

Step Two: Use Traditional Utensils Commonly Used by Community Members

The importance of choosing utensils is that they are representative of common use among members of society, which plays an active role in the success of the food atlas (as it can affect the quantity consumed), which in turn affects consumers’ choice of the correct estimated quantity.

- Therefore, it is important to identify traditional and commonly used tools to improve the accuracy of the Food Atlas assessment.
- It is difficult to identify specific tools to build a comprehensive food atlas based on reliable scientific sources. On the other hand, after reviewing the experiences of other countries in creating food atlases, there are several specific criteria that help in choosing the utensils that are commonly used by members of society to be represented in the food atlas, including the following:

1. Use a white plain flat plate of medium size for main dishes and all other types of foods (serving plate for one person with a diameter of 24 cm).
2. Use a small deep white bowl for different types of soups and similar foods (serving bowl for one person with a diameter of 18 cm).
3. Place a spoon, a fork, a knife, or all of them next to the plate to facilitate the accurate assessment and estimation of the quantity of food consumed for users of the food atlas.
4. Indicate more than two food items in the same dish at once, unless necessary, to facilitate the process of estimating consumption and to stop distracting the user of the food atlas.
5. Use a clear glass cup for water and all other drinks (a glass cup of 250 ml).

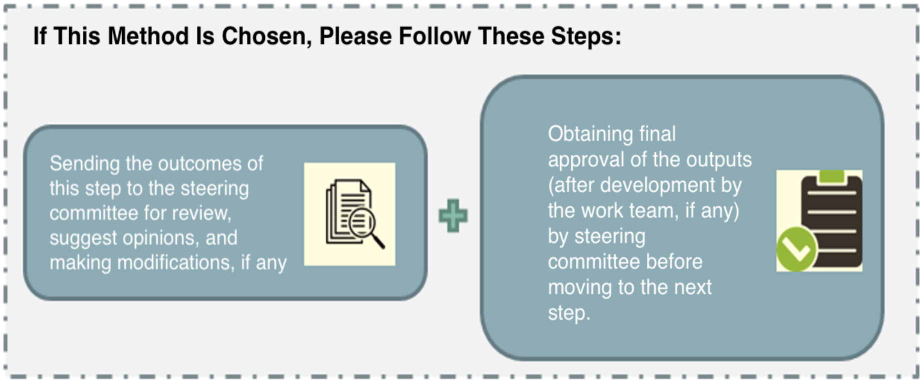

Step Three: Determine the Portion Size

- The size of the food portion is determined based on the usual food intake of the population.
- The average portion size is chosen in such a way that it is representative of the actual amount consumed by individuals in the community.

<u>A: If the information about the portion size is available:</u>

– Gather information about food intake through questionnaires, national surveys, or reliable scientific studies that contain food and beverage items, as well as quantities.

<u>B: If the information about the portion size is not available:</u>

1. Estimate the usual consumption of the population using information obtained from the steering committee.
2. After choosing the most appropriate method to determine the portion size consumed by individuals, the weights of the quantities of food consumed are measured directly using electronic scales to the nearest 0.1 kg, and to the nearest 0.1 ml for liquids.
3. <u>It is preferable</u> to specify three to six different food portions for each type of food that have been previously identified.
4. When choosing to add three food portions for each food item, the weights of the actual portions could be determined to show the weights of the portions between each two portions.
5. Display food portions from smallest to largest.
6. Display food portions for each type of food on the same container to avoid the misinterpretation of the portion sizes.
7. Denote each food portion by a letter or number.

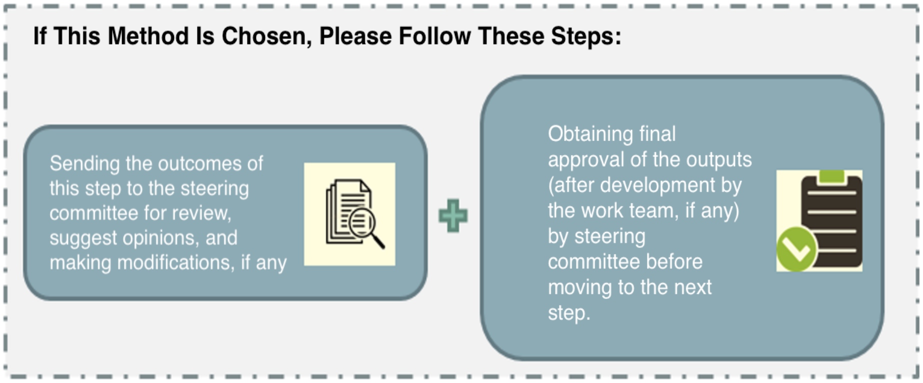

Step Four: Food Photographs

- One of the most important challenges when designing and photographing a food atlas is trying to minimize error in portion size estimates. Among the factors affecting the possibility of increasing error in determining serving size are the following:

Size of image

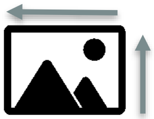

1. A single A4 photograph (20 x 29 cm) to eight (6 x 8 cm) images put on one A4 page.
2. Choosing eight (6 x 8 cm (A7)) images is the best format as it provides the largest amount of useful information in the least amount of space.

Portion sizes

3. Choose an even number of photographs (either four, six or eight), and avoid odd numbers because subjects may be tempted to choose the central image.
4. Choosing more images (e.g., eight vs. four) results in more accuracy.

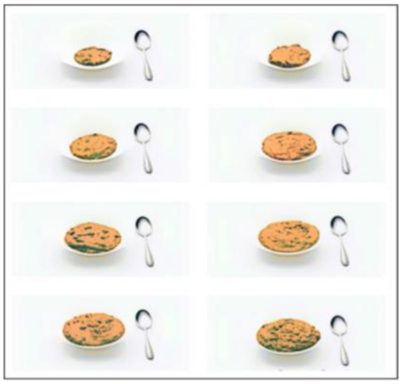

Image (1)

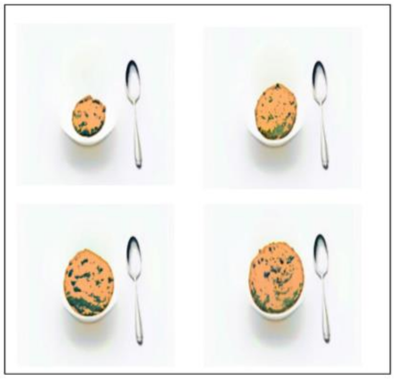

Image (2)

Interval

5. A large interval will result in loss of information about actual amounts consumed.
6. A very small interval may yield images so similar in appearance that subjects are unable to distinguish between them, leading to frustration and loss of attention. For greatest precision, it is desirable to find an interval that is just at the limit of subjects' ability to distinguish amounts depicted in adjacent images.

Order of presentation

7. Present the images from smallest to largest portion size in every photograph series.

Labels

8. Label the images with numbers or letters.
9. The label should be clear but not too large or conspicuous as to obscure or distract from the appearance of the food in the photograph. See image 3.

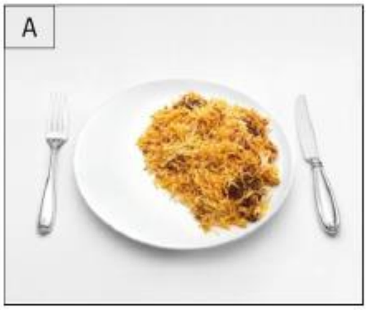

Image (3)

Background

10. The background should be unobtrusive and neutral in character.
11. Reference objects (e.g., plate. knife and fork or other cutlery) should be included in every photograph and provided to subjects as real objects or life-size photographs.

Color

12. Choose any color for the images; there is no difference between colored or black and white photographs in the occurrence of errors in the estimation of portion size.

Camera

13. Use a large, diffused strobe flash to provide soft lighting rather than atmospheric or contrasting lighting in order to maximize the clarity of the food.
14. Use a professional camera with a standard lens to give the same perspective as the human eye.

15. The camera's height of view should mirror that of a person of average height sitting at the table looking at the plate of food on the table in front of them.

Step Five: Validation of Food Atlases

- The validation process of food atlas plays a critical role as it provides confirmations that the developed food atlas is valid to use.

Visual estimation of food atlas amount

1. Select a randomized sample from the public to participate in the validation process.
2. Randomly select 15% of the total food items included in the atlas and prepare them for the participants.
3. Prepare open questions for the participants to answer about the design of the food atlas, arrangement, photo clarity, and food items.
4. Identify the prepared dishes with a code and use another code to define different portion size (making sure that the code did not provide any clues to the size of the portion).
5. Assign each participant with one photo for each food item (The participant should cover each portion size of different food items [1 food item → 1 portion size]).
6. Ask the participant about specific reference for his food items and estimate the portion size by two different way:
    a. First, make the participant select the image of reference from the prepared dishes.
    b. Second, make the participant select the image of reference in the photographic atlas.
7. Statistically analyze the comparison between the estimations of both methods for each participant.
8. Analyze the prevalence of correct image selection.

Recall foods using food atlas photographs

1. Select a randomize sample from the public to participate in the validation process.
2. Make the participants select 30% of the total food items that are included in the atlas (conditionally choosing food dishes that are easily and usually made at home).
3. Select 15% of the total food items from the list that participants chose before.
4. Prepare open question for the participants to answer about the design of the food atlas, arrangement, photo clarity, and food items.
5. Prepare the dishes and weigh them on a scale.
6. Ask the participant to estimate the portion size in two different ways:
    a. First, let the interviewer ask the participant to compare the presented food with the photographs in the atlas.
    b. Second, give the participant a scale (identical to the one used before) to take home.
        i. Let the participant weigh of all foods consumed at one meal and recall the amount of food eaten the previous day using the photographic atlas.
7. Statistically analyze the comparison between the estimations of both methods for each participant.
8. Analyze the prevalence of correct image selection.

- While the committee comments on the content and the edits are being made, the principal researcher and the work team have the right to re-verify the validity of the atlas by repeating the previous step again or adopting the committee's comments after amending them and proceeding to the next step.

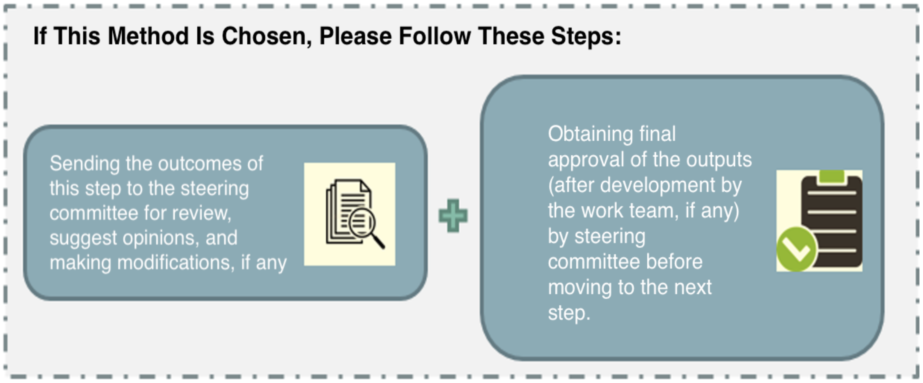
If This Method Is Chosen, Please Follow These Steps:
Sending the outcomes of this step to the steering committee for review, suggest opinions, and making modifications, if any
Obtaining final approval of the outputs (after development by the work team, if any) by steering committee before moving to the next step.

Step Six: Food Atlas

After validation and approval of the atlas are carried out in the previous validation steps by the Steering Committee and the working team, the atlas is published as a hard copy or electronic version based on the available resources and the accuracy of the working team's plan.

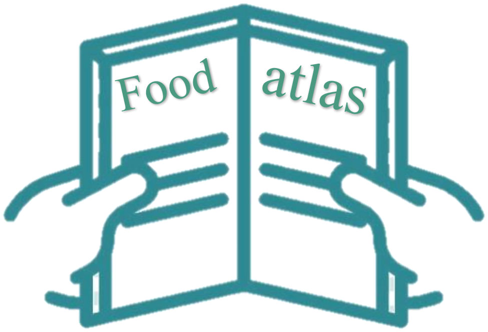

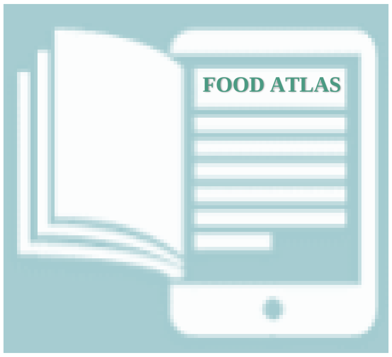

Supplement: Supplementary file 2 [file Data_Sheet_1.pdf]
